# The Power of Customized Clear Aligners in Closing Molar Edentulous Spaces: Clinical and Medico-Legal Considerations in a Scoping Review and Case Report

**DOI:** 10.3390/jpm13091389

**Published:** 2023-09-16

**Authors:** Alessandra Putrino, Enrico Marinelli, Simona Zaami

**Affiliations:** 1 Department of Anatomical, Histological, Forensic and Orthopedic Sciences, Sapienza University of Rome, 00161 Rome, Italy; alessandra.putrino@gmail.com; 2 Department of Medico-Surgical Sciences and Biotechnologies, Sapienza University of Rome, 04100 Latina, Italy; enrico.marinelli@uniroma1.it

**Keywords:** orthodontic space closure, tooth extraction, clear aligner appliances, medico-legal aspects, professional liability

## Abstract

Successful closure of edentulous spaces with clear aligners (CAs) is influenced by many factors. CAs are tailored orthodontic devices whose predictability may have relevant medico-legal implications. This study presents a scoping review about missing molar space closure (MMSC) with CAs and a clinical case. This study aims to highlight the feasibility of molar space closure by mesialization with CAs without hybrid supports. Following PRISMA Sc-review guidelines, English-written randomized/non-randomized/observational clinical studies on PubMed, Scopus, Cochrane and Lilacs were searched. An 18-year-old patient, with upper and lower edentulous spaces due to the loss of two first molars, was rehabilitated with CAs (Sorridi^®^, Sorridi srl, Latina, Italy) without hybrid supports and attachments. The therapy was carried out over 10 months. Currently, there are no studies documenting MMSC by mesialization with only CAs. Existing articles document the closure of premolar or incisor spaces. The upper and lower left second molars replaced the missing first molars, and erupting third molars replaced adjacent teeth. The biomechanical effects in space closure with CAs related to extraction cases appear as priorities of clinical/medico-legal interest. Our case turns attention to this movement of CAs without attachments/hybrid supports, indicating that even such a complex treatment can be comfortable for patients and safely predictable for specialists.

## 1. Introduction

Treatments with clear aligners (CAs) have become very popular over time, thanks to their comfort, esthetics and improved oral hygiene [1,2,3]. Compared to fixed appliances, they have a different biomechanical system, which affects their ability to obtain programmed dental movements. Final corrections may be necessary depending on the ability of the viscoelastic polymer material to transfer adequate and effective forces [4,5]. Alignment of anterior sectors, closure of diastemas and premolar distalization are movements in which CAs have proved to be effective solutions. Correction of deep bites, rotations, arch expansions and movements that require root control are more likely to be unsatisfactory [6,7,8]. For this reason, in extractive cases, hybrid systems that offset the insufficient biomechanical performance at the pure tooth–aligner interface are preferable [9,10,11,12]. Orthodontic forces are attenuated by wearing CAs that disallow them from exerting sufficiently constant strength and elasticity without creep and stress relaxation [13,14]. Tooth size, morphology and dental arch shape affect CA’ adhesion to the tooth and retention. Movement strategies supported by composite buttons, called attachments, may not be effective or predictable in complex movement patterns, even when customized [6,15]. Mini-screws are temporary anchorage devices widely used in hybrid treatments with CAs to assist anchorage control during extraction-space closure [16,17]. Minimal corticotomies, intentional injuries to the cortical bone, can also facilitate extraction-space closures in patients undergoing orthodontic therapies with CAs [18,19]. Both approaches, mini-screws and corticotomies, although considered safe, are not always easily accepted by patients. They are often perceived as invasive methods, as opposed to the comfortable protocols expected from therapies with CAs [20,21,22,23]. The experiences reported in the literature on complex cases treated with hybrid therapies for extraction-space closures refer exclusively to traditional systematics proposed by the trademark founder of CAs (Invisalign^®^, Align Technology, Inc., Tempe, AZ, USA). This system, which due to its characteristics, is very different from more recent, but still inconclusively documented, CA systems [6,9,13]. The predictability of movements achievable with orthodontic devices is very important. Clinicians can pre-emptively evaluate therapeutic feasibility. Through CA setups, patients have a tool for perception and awareness of what may be their expectations for treatment results [3,7,24]. At a time when therapies are increasingly personalized and tailored to each patient’s multifactorial characteristics, and the devices used are highly customized, such aspects should not be underestimated. From a medico-legal standpoint, orthodontists have a duty to guarantee the best possible treatment and the most realistically achievable result [25,26]. Orthodontic practice risk is of particular interest because it encompasses both the esthetics of the smile and the masticatory function [27]. After a scoping review on missing molar space closures with CAs, the objective of this study is to elaborate on a case of maxillary and mandibular closure of edentulous spaces for the loss of two permanent first molars in a young adult patient. The patient refused rehabilitation with prosthetic implants and orthodontic treatments with fixed or hybrid fixed-removable therapy. She was therefore treated with a system of CAs without attachments and without the aid of hybrid strategies.

## 2. Materials and Methods

### 2.1. Scoping Review

A preliminary review of the literature on the closure of edentulous extractive spaces of molars was carried out by consulting the main scientific literature databases (PubMed, Scopus, LILACS, Cochrane). This scoping review, developed on the basis of the PRISMA-ScR (Preferred Reporting Items for Systematic reviews and Meta-Analyses extension for Scoping Reviews) guidelines [28], had to answer the research question “do orthodontic patients under orthodontic therapy with clear aligners have efficient edentulous space’s closures when upper and lower molars are congenitally missing or lost for disease?”. This question was formulated according to the acronym PCC: population/problem, concept, context (Table 1). The search was conducted using the following MeSH terms and free terms in combination with the Boolean operators “AND” and “OR”: clear aligners, space closure, extraction, missing and molars. This scoping review project has now been (29 March 2023) registered as an OSF-Standard Pre-Data Collection Registration on OSF Registries (Open Science Framework, Center for Open Science©, 2011–2023). The research took place on 30 and 31 July 2023 following the eligibility criteria that were established as follows. Inclusion criteria for the review belonged to the categories of randomized, non-randomized and observational studies, documented with articles that can be consulted in both the abstract and in the full-text reading, and written in English. No limits on the year of publication were placed. Case reports and case series, commentaries and letters to editors, reviews on the subject and articles not written in English were excluded. In vitro, animal or finite element analysis studies were also discarded (Table 2). The initial results of the various databases, whose duplicates were eliminated thanks to the Zotero software (Zotero 5.0 for Windows, Corporation for Digital Scholarship, Vienna, VA, USA), were then selected on the basis of the availability of the abstracts and the verification that the contents, then deepened by the full-text reading, met the pre-established eligibility criteria until obtaining the final number of resources to be included in the discussion of the results, subjected only to descriptive statistical analysis. Since the revision was performed by two experienced orthodontists with decades of experience, all phases were carried out autonomously, and only at the end were the results combined and compared. Any possible discordant opinions or studies subject to dubious classification were overcome through a shared review of the specific content and full-text readings of the results excluded from the primary examination of the abstract. A third operator, also a specialist in orthodontics, supervised the review process. The contents of any included studies are charted according to the authors, year of publication, study design, sample characteristics (age, sex, distinction into groups), type of intervention and outcome.

### 2.2. Case Report

**Figure 1:**
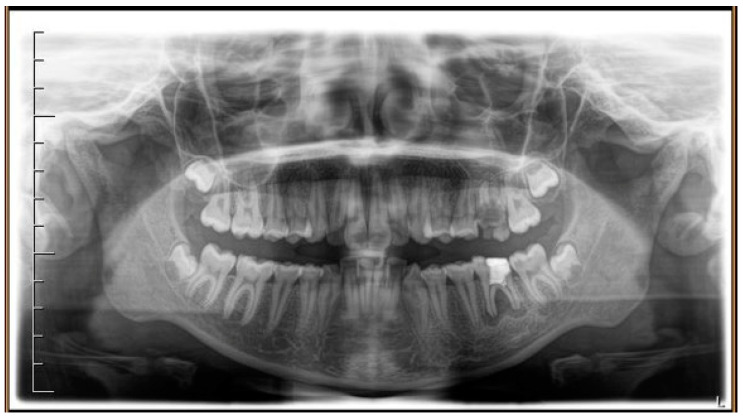
Pre-treatment radiograph of the patient. The first two left molars appear irreparably compromised by destructive caries.

**Figure 2:**
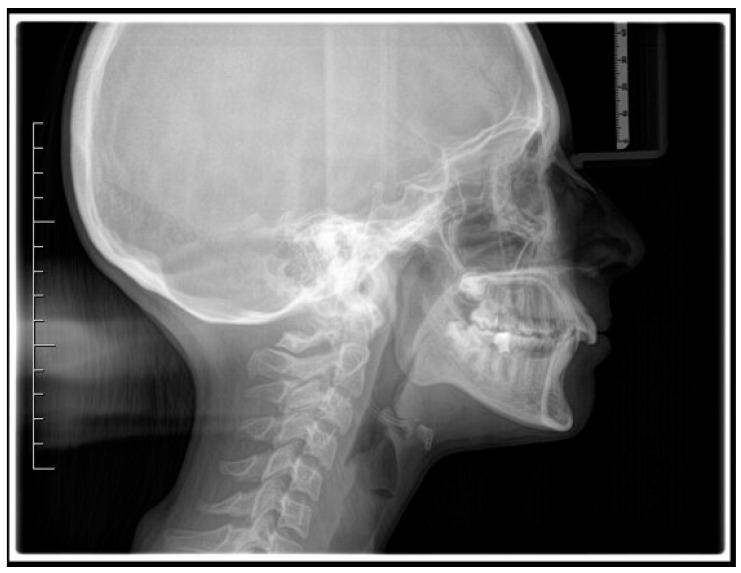
Pre-treatment lateral teleradiography.

**Figure 3:**
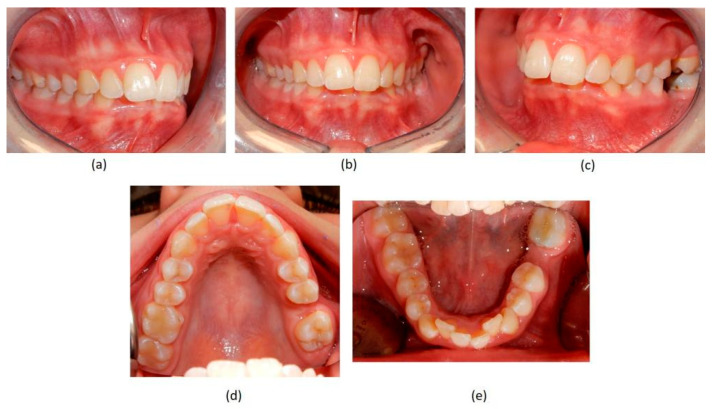
Pre-treatment intraoral photographs. (**a**) right-side view; (**b**) deep bite in frontal view; (**c**) absence of the two first molars on the left side; (**d**) upper occlusal view showing the absence of the left first molar; (**e**) lower occlusal view showing the absence of the left first molar and the moderate crowding of the incisors.

A case of CA therapy without attachments and without the aid of hybrid elements has been documented. The patient, an 18-year-old woman at the beginning of treatment (October 2021), signed written informed consent to orthodontic treatment and authorized the use of her anonymized treatment data, including photos, X-rays and virtual models of the dental arches. She needed to rehabilitate two edentulous areas corresponding to first maxillary and mandibular molars, both on the left side, assessed as irrecoverable for destructive caries at the time of the orthodontic evaluation (Figure 1). The patient refused rehabilitation with prosthetic implants and orthodontic treatments with fixed or hybrid fixed-removable therapy. Orthopanoramic (Figure 1) and lateral teleradiography of the skull (Figure 2) were acquired to make the necessary assessments. The cephalometric examination was performed using the Cephio© Cephalometric Analysis artificial intelligence driven platform (Cephio sp. z. o. o. [Ltd.]). Her skeletal class II malocclusion was due to maxillar and mandibular retrusion (SNA 80°, SNB° 74.6°, ANB 5.4°, SNPog 76.2°). She had “long face” (OP-SNP 21.1°), a posterior inclination of the jaw (MPGoGn- SNP 35.4°), slight hyperdivergence (FMA 26.2°), with pro-inclined lower incisors (1-NB 28.4°, IMPA 96.1°) and retroclined upper incisors (1-NA 14.1°, FMIA 57.7°). Overjet and overbite were severely altered (6 and 7 mm) (Table 3). Orthodontic treatment started 4 months after the first left maxillary and mandibular molar extractions. Intraoral and facial photos were captured (Canon© Coolpix A900, Canon, Tokyo 146-8501, Japan). Dentally, she had a first molar and canine class on the right side, and a first canine class on the left side in which the molar class was no longer assessable (initially it was a first class); however, a partial reduction in edentulous spaces was observed due to the movement of adjacent teeth (Figure 3) and lower incisors were moderately crowded. Precision silicone impressions (Elite HD+ Putty Soft Normal and Elite HD+ Super Light Body, Zhermack SpA, Badia, Italy) were taken using the double technique. Then, a virtual setup of the treatment with CAs was developed to evaluate its feasibility. It showed full closure of edentulous spaces and was also discussed successively with the patient. The orthodontist clarified there were no data from the literature (at that time) to support the successful movements required with only CAs. The orthodontist and patient started treatment by agreement. The informed consent to treatment was signed. It also covered clinical re-evaluation with solutions less favorable to the patient’s preferences if the results in the middle of treatment had been clinically unsatisfactory or unfavorable. The orthodontist explained the need to intercept any treatment complications to the patient. This also related to the predictability of intermediate results for medico-legal reasons, for the mutual protection of her as a patient and of the orthodontist as a professional responsible for the therapy. Initial and final virtual models were superimposed to evaluate the movements obtained (Maestro 3D©, AGE Solutions S.r.l., Pontedera- Pisa, Italy). At the end of the therapy, new X-rays were requested and new intraoral and extraoral photos were recorded.

## 3. Results

### Scoping Review

**Figure 4:**
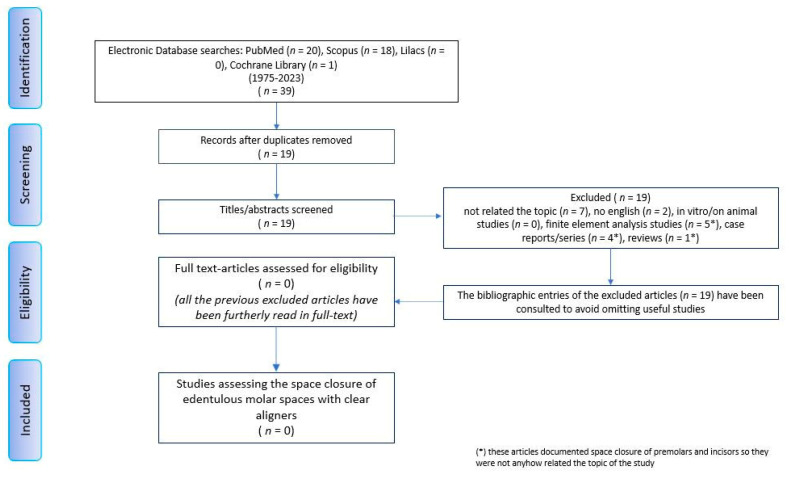
Prisma flow chart diagram.

The database search initially yielded 39 results (PubMed 20, Scopus 18, Cochrane Library 1, LILACS 0) published between 1975 and 2023. Duplicates were removed using the Zotero software. The remaining 19 results were checked for eligibility criteria by reading the abstracts. Following this phase, all 19 articles were discarded since they did not meet the established eligibility criteria. Seven articles were excluded because their topic was different (premolars/incisor extraction site closure by mesialization of the anterior teeth with removable or fixed appliances including clear aligners); two articles were written in the Chinese language; five articles documented finite element analysis (related to premolar space closures), four articles presented case reports/series (related to premolar extraction site closures by clear aligners and hybrid systems); one article was a systematic review (related to premolar space closures with clear aligners and a hybrid approach). The bibliographic entries of all the articles were consulted to look for any further studies suitable for our aim. No other studies emerged from this supplemental research (Figure 4). It should be noted that although there were no studies of dubious exclusion, full-text reading of all the articles was performed for further verification at the end of the source’s selection flow; nevertheless, it unequivocally confirmed the non-relevance of the content with the objective of revision regardless of the design of the study. The comparison of reviewers’ reports has definitively established the lack of useful results for the topic of this review.

**Figure 5:**
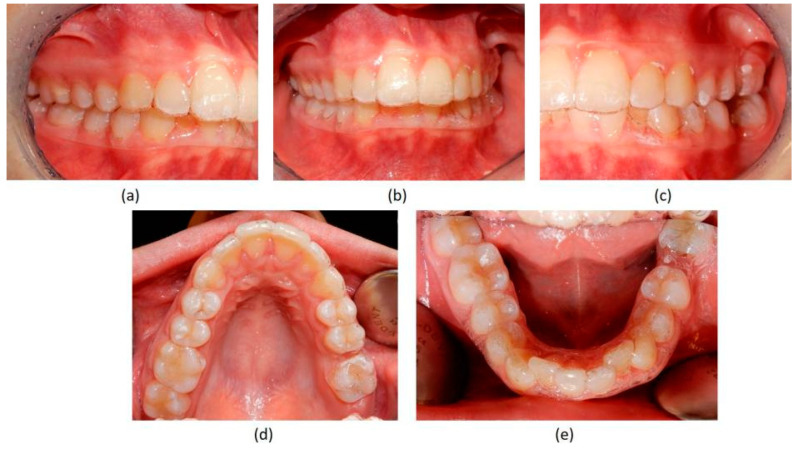
Control photo with soft aligners number 9 as required by the company, Sorridi, to monitor the good progress of the therapy. (**a**–**e**) views show progressive closure of spaces.

**Figure 6:**
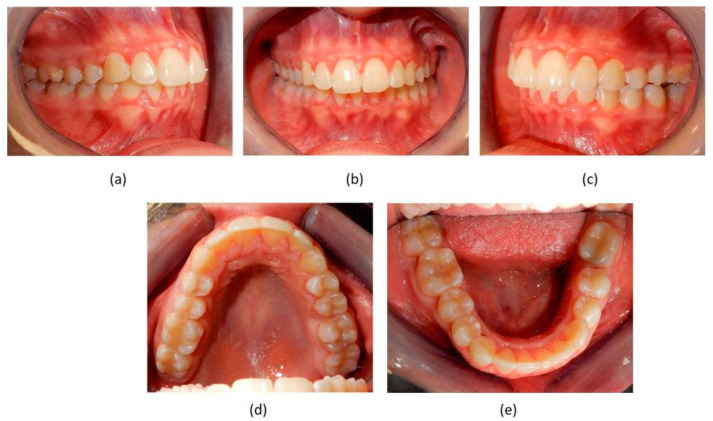
Post-treatment intraoral photographs. (**a**–**c**) views show final occlusion; (**d**) Upper extractive space closed on left side by mesialization of second molar in the place of first missing molar; (**e**) remaining slight mesio-rotation of second left molar, orthodontically transposed in the missing first molar space, but extractive space was efficiently closed.

**Figure 7:**
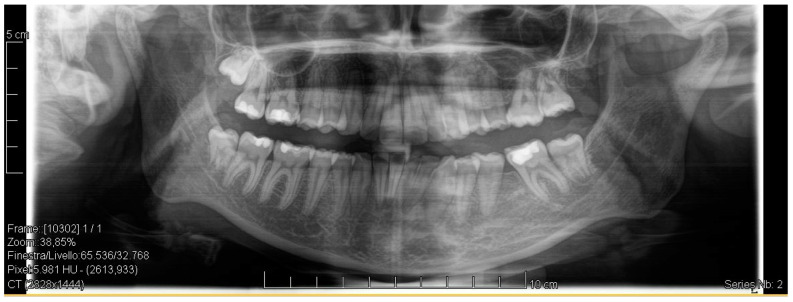
Post-treatment radiograph showing extractive spaces’ closure.

**Figure 8:**
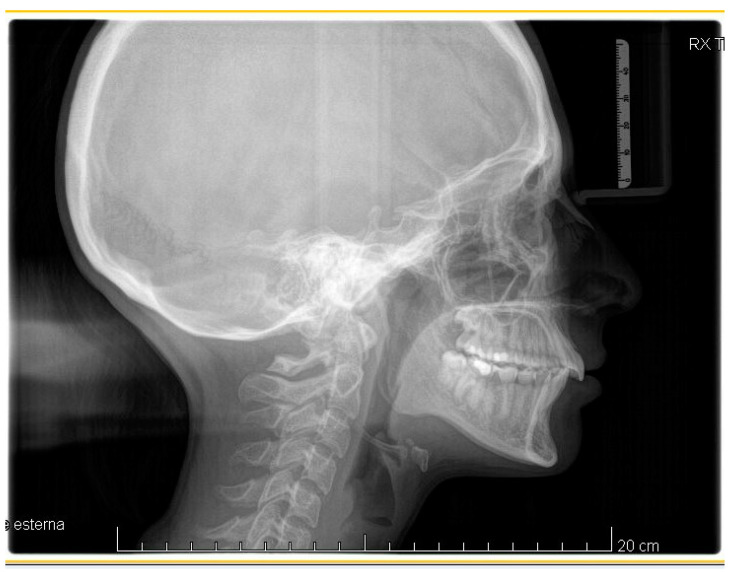
Post-treatment lateral teleradiography.

**Figure 9:**
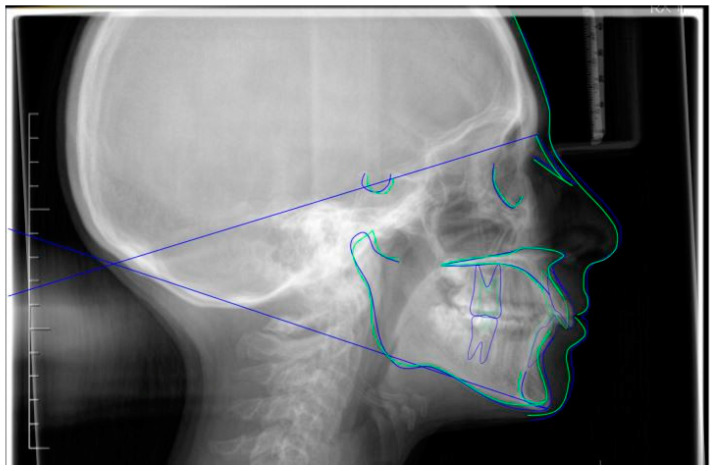
Superimposition of pre- (blue) and post- (green) cephalometric traces (reference plane SN) with appropriate function on the Cephio platform.

**Figure 10:**
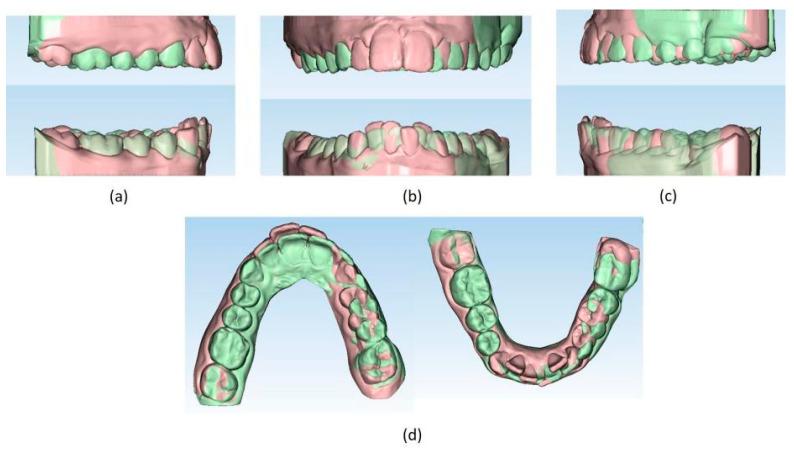
(**a**–**d**) Superimposition of pre- (light orange) and post- (light green) treatment virtual models (Maestro 3D© software, Version 6, AGE Solutions S.r.l. Pontedera, Tuscany, Italy).

The setup indicated the need to treat the patient with 44 upper and 24 lower aligners, equal to 22 movements in the upper arch and 14 movements in the lower arch. The treatment required 10 months and 2 weeks. The Sorridi^®^ system (Sorridi^®^, Sorridi s.r.l., Latina, Italy) used in this clinical case is a no-attachment system, which alternates weekly between two aligner thicknesses: soft (0.06 mm) and hard (0.08 mm), for each programmed movement. Soft aligners are used in the first week, then hard aligners are used for another week until the next soft-type aligner. The gingival margin is straight beyond the gingival zenith above 2 mm (Figure 5). No interproximal reduction (stripping) and divots to guide dental movements were required. No hybrid supports were programmed (corticotomies, mini-screws, elastics). No further refinements were deemed urgent. Orthodontic retainers were applied even if a slight mesio-lingual rotation of the second left lower molar orthodontically transposed in place of the missing first molar was present (Figure 6). Post-treatment radiographs were requested (Figure 7 and Figure 8) and new cephalometric analysis was performed (Table 3). The patient finished the treatment remaining in the skeletal class II from biretrusion (SNA 78.5°, SNB° 73.8°), but the unfavorable ratio between maxilla/mandible improved (ANB 4.8°). The long and hyperdivergent face pattern remained (OP-SNP 21.6°, FMA 27.5°). The mandibular inclination returned to normal values (MPGoGn-SNP 34°). Lower incisors’ inclination was corrected (1-NB 25.4°, IMPA 94.8°), upper incisors’ inclination strongly improved (1-NA 17.4°) and overjet and overbite were strongly reduced to values almost close to normal (both equal to 3 mm) (Figure 9). Pre- and post-treatment superimposition of virtual models allowed us to evaluate the extent of movements that were actually obtained (Figure 10). Furthermore, distances were measured on virtual models before and after the orthodontic treatment. The upper edentulous space (from the distal surface of the second premolar to the mesial surface of the second molar) initially measured 5.33 mm and 0.11 mm at the end of treatment, the lower one (distal to the second premolar and mesial to the second molar ipsilaterally) was 7.68 mm and it was closed completely, leaving just 0.9 mm. The distance between the cusp of the left upper canine and the disto-buccal cusp of the ipsilateral upper second molar was 29.88 mm, and then decreased to 24.33 mm with a variation in the upper intercanine distance of just 1.64 mm (from 28.31 to 29.95 mm). It indicates that much of the closure was due to mesialization of the molar as well as distalization of the lateral teeth. In the lower arch, the distance between the cusp of the left canine and the disto-vestibular cusp of the second molar decreased, going from 30.42 mm to 24.59 mm, almost 6 mm, with a variation in the intercanine distance from the initial 20.36 mm to 23.73 mm. The resolution of anterior crowding and a reciprocal movement of distalization of the canine-premolar sector and mesialization of the second molar, all equal to about half a cusp each, led to an optimal closure of the posterior edentulous space.

## 4. Discussion

CAs are in great demand even for cases considered complex by expert orthodontists [1,2,5,7]. Their popularity is linked to esthetics and convenience for the patient [1,3]. During the COVID-19 pandemic, aligners allowed simpler remote controls, faster control sessions and good performance of therapies despite restrictions and lockdowns [29,30,31]. However, a major problem with CAs lies in their predictability for carrying out certain types of tooth movement [4,7,8]. Virtual setups generated through therapeutic planning are often not realistic about the duration of treatments. A succession of refinements is often required to complete cases successfully [32,33,34]. Furthermore, biomechanical effects conditioned by factors, such as the materials used and design of aligners, are highly variable [4]. There are very different aligner systems for clinical use whose effects are not yet well-documented [6,8]. These predictability considerations have a legal relationship with the medico-legal dimension of guaranteeing results in orthodontics [35]. However, the international scientific literature is decidedly oriented towards describing the system which introduced aligners to the market. Its main characteristics (scalloped margin, presence of attachments, single thickness) do not match alternative ones (straight and high margin, absence of attachments, divots) that are increasingly common [6]. No mention is made of the medico-legal implications related to the use of devices classified as equal, but in reality profoundly different [4,6,7,27,35]. The first-generation aligner systems have introduced hybrid systems (elastics, mini-screws, corticotomies, etc.) to support complex movements scarcely achievable with a pure system [9,10,13,16,17,18,19,20,21], but little is known about other systems in both pure and hybrid forms [6,11,12,36]. Thus, the attempt to find data relating to MMSC through the reciprocal movement of mesialization of other molars present and the distalization of latero-posterior teeth, brings out an important fact: this movement has not yet been described with any CA system [37]. Various types of studies available in the literature document the closure of posterior dental spaces due to extraction of premolars (mostly upper) by retraction of the anterior sector [38,39,40]. One of these studies focuses treatment on torque management in a patient with a skeletal class II relationship in which extractions of the first maxillary premolars were performed. The case was resolved in 52 months using CA treatment supported by palatal mini-screws and a modified double J retractor. Placing the line of action higher rather than at the center of resistance allowed for good torque control [38]. In another study, four cases were presented on adult patients with a skeletal class II relationship requiring the extraction of the first maxillary premolars. They underwent therapy with aligners and mini-screws inserted on the vestibular or palatal side. Spaces were closed in 16–25 months with good torque control [39]. An older prospective study compared the ability to complete a series of CAs in fifteen patients without interference or problems, and whether they needed extractions of maxillary premolars. They were treated with aligners with two different thicknesses (soft and hard) and a weekly or biweekly change. Their results suggested a better course of therapy with a biweekly change and without extractions [40]. Only one case report dealt with our topic of closing extraction spaces through molars using CAs. Minimally invasive corticotomies were used, but the data presented are somewhat lacking and do not allow for any comparisons [18]. With one of the patients (missing first right upper molar, lower molars and lower moderate crowding), it was planned to close their upper space by distalization of the first quadrant and mesialization of the second and third right upper molars. The opening space was used to insert a dental implant at the first right lower-molar site. They used CAs and optimized attachment on the second right upper premolar to achieve distalization and provide anchorage for molar mesialization. Interdental corticotomy was performed in the first quadrant (from the distal site of the central incisor to the distal site of the second molar) and in the third quadrant (from the distal site of the lateral incisors to the mesial site of the first premolars and between the central incisors). The case required 9 months and 3 weeks. Even if CAs were not reported, the authors viewed corticotomy as an advantage, due to a reduced treatment duration and lower long-term risk of relapse. However, clinical outcomes are not well-documented. In our experience, it is possible to achieve the closure of extraction spaces in much less time (10 months versus 16–52 months) without hybrid strategies. The reciprocal movement of molar mesialization and distalization, involving the premolars, may suggest that the retraction movement of the anterior sector with a systematic without attachments, with straight and high margins, and differentiated thickness with weekly change is quite likely. In our case, the upper arch underwent an ipsilateral distalization to the extraction site both at the maxillary and mandibular level. A reduction in the excessive inclination of the upper and lower incisors was observable. No undesirable effects were detected to suggest that the use of mini-screws, elastic bands or other devices could have played a protective action. This can be explained precisely by the different systematics used compared to the traditional ones. In a case report on hybrid therapies between CAs and temporary anchoring devices (TADs or mini-screws), we read that “when using clear aligners, if distalization greater than 3 mm is required, there is no real predictable procedure to follow… Clear aligners are effective at controlling upper molar bodily predictable movements of about 1.5 mm to 3 mm” [17]. Previous experiences describe distalization of maxillary molars as a safe movement without the risk of undesired tipping and intrusion. For this bodily movement beyond 2.25 mm, it would be necessary to undertake hybrid strategies [41]. The literature on movements with aligners still lacks evidence regarding the opposite movement, the mesialization of molars, documented in our study. Therefore, in terms of predictability, this movement raises the patient’s expectations for success and thus places that extra onus on the professional [42,43,44]. Although signed informed consent provides a degree of protection for doctors, the lack of unambiguous standards and guidelines on the subject puts them in a potentially difficult position in case of litigation stemming from malpractice charges [43,45]. Malpractice is regulated in many countries, so if good practice guidelines are not provably complied with, the risk of negligence malpractice charges is real in the case of adverse outcomes [46,47].

## 5. Conclusions

The promising results achieved in the case herein reported match those expected in the virtual setup. Although the outcome is encouraging, it leads us to reflect on the medico-legal importance of dental movement reliability, especially with customized devices like CAs. It would be important to have further clinical data on the limitations and potential with as many as possible innovative CA systematics available. Such findings could confirm or refute what happens with traditional first-generation systematics described in the literature for simple and complex movements. The complex closing movements of edentulous spaces in first-generation aligners would require the aid of hybrid strategies that are not always well accepted by patients. CAs are in fact perceived as more comfortable and less invasive devices than other solutions. However, this cannot supplant patients’ expectations of obtaining satisfactory results, nor can it exempt the specialist from the professional obligation to use the most predictable devices possible just because they want to please patients.

## Figures and Tables

**Table 1:** Research question based on the PCC (Population/Problem, Concept, Context) strategy.

Population/Problem	Orthodontic Patients
Concept	Clear aligners efficiently close edentulous spaces
Context	Congenitally missing or lost for diseases upper and/or lower molars

**Table 2:** Inclusion and exclusion criteria.

Inclusion Criteria	Exclusion Criteria
Randomized and non-randomized clinical, observational studies	In vitro and in vivo (animal) studies, finite element studies, case reports/case series, reviews, commentaries, letters to editors
English language	Other languages
Abstract and full-text reading available	No abstracts and/or full-text reading

**Table 3:** Pre- and post-treatment cephalometric analysis.

Cephalometric Landmarks	Pre-Treatment	Post-Treatment	Normal Values
SNA	80°	78.5°	82 ± 2°
SNB	74.6°	73.8°	80 ± 2°
ANB	5.4°	4.8°	2 ± 2°
OP-SNP	21.1°	21.6°	14 ± 2°
MP(GoGn)-SNP	35.4°	34°	32 ± 3°
+1- NA	14.1°	17.4°	22 ± 2°
+1: NA (mm)	4.1 mm	4.8 mm	4 ± 2 mm
−1- NB	28.4°	25.4°	25 ± 2°
−1: NB (mm)	4.7 mm	5 mm	4 ± 1 mm
Interincisal Angle	132.1°	132.4°	131 ± 8°
FMA	26.2°	27.5°	22 ± 5°
FMIA	57.7°	57.7°	68 ± 7°
IMPA	96.1°	94.8°	90 ± 5°
SNPog	76.2°	75.6°	81 ± 3°
Saddle Angle (N-S-Ar)	125.1°	122.6°	123 ± 5°
Articular Angle (S-Ar-Go)	142.5°	145.9°	143 ± 6°
Gonial Angle (Ar-Go-Me)	127.8°	125.5°	130 ± 7°
Upper Gonial Angle	53.3°	53.4°	52°–55°
Lower Gonial Angle	74.4°	72.1°	70°–75°
Bjork’s sum	395.4°	394°	396 ± 6°
Anterior Cranial Base (N-S)	59.9 mm	63 mm	71 ± 3 mm
Posterior Cranial Base (S-Ar)	29.2 mm	32.2 mm	32 ± 3 mm
Ramus Height (Ar-Go)	37.1 mm	35.1 mm	44 ± 5 mm
Mandibular Body (Go-Gn)	61.1 mm	64.6 mm	71 ± 5 mm
Posterior Face Height (S-Go)	62.8 mm	64.4 mm	70–85 mm
Anterior Face Height (N-Me)	100.2 mm	103 mm	105–120 mm
Facial Height Index	61.5%	61.5%	Clock < 65; Anti-clock > 65
Upper Lip	0 mm	0 mm	0 mm
Lower Lip	2.4 mm	2 mm	0 mm
Overjet	6 mm	3 mm	2.5 ± 2.5 mm
Overbite	7 mm	3 mm	2.5 ± 2.5 mm

## Data Availability

Not available.
